# Empowering women to take control over cervical screening

**DOI:** 10.1016/j.lanwpc.2021.100339

**Published:** 2021-11-30

A study published in *The Lancet* on Nov 2, 2021, shows that the introduction of the human papillomavirus (HPV) immunisation programme in England, UK has led to an 87% relative reduction in cervical cancer cases in women who were vaccinated when they were aged 12–13 years. Cervical cancer is the fourth most common cancer in women and most cases are caused by HPV infection. WHO estimated that 604 000 new cases and 342 000 deaths due to cervical cancer occurred globally in 2020. In the Western Pacific region, approximately 193 000 women were diagnosed with cervical cancer and 74 900 women died from the disease in the same year. Among these deaths, nearly 90% occurred in low-income and middle-income countries (LMICs).

The *Lancet* study confirms that with the implementation of highly effective prevention measures, including the HPV vaccine and cervical screening, cervical cancer is now a preventable disease. However, these prevention strategies are not equitably executed across and within countries in the Western Pacific region.

In parallel to the push for more widely accessible HPV vaccines in the region, existing cervical screening services should be strengthened and recognised as a priority. Pap smears and visual inspection have been the standard screening methods in many countries, which have effectively reduced the burden of cervical cancer in many high-income countries. Even though almost all countries in the Western Pacific have national screening programmes for cervical cancer, screening coverage is relatively low in LMICs as of 2019. In addition to the health system-related barriers that hamper the delivery of this crucial service, negative emotions towards screening, cultural or religious taboo and stigma, lack of time, and cost are common reasons for women to not attend cervical cancer screening. In countries where good medical services are available, women might still not get screened regularly. Indigenous women are more likely to die from cervical cancer than their non-Indigenous counterparts in Australia due to a low screening rate. In *The Lancet Regional Health – Western Pacific*, Rosalind Moxham and colleagues’ qualitative study shows that Indigenous women have different attitudes and behaviours towards cervical screening. Instead of having a uniform executive plan, the study suggests that targeted and ongoing campaigns about cervical screening are imperative for this population.

Recent cervical screening guidelines have added HPV testing to primary screening. HPV testing has an effectiveness that is equivalent with or superior to cervical cytology. Besides, it has the potential to simplify sample collection via self-swabbing. In a community trial published in this issue of *The Lancet Regional Health – Western Pacific*, Naomi Brewer and colleagues find that home-based self-sampling increased the participation rate in cervical screening of never-screened and under-screened women in New Zealand. Increasing the participation rate for cervical screening in Asian, Māori, and Pacific women from New Zealand could reduce demographic and social inequities that pose barriers to cervical screening for these underserved women. Self-sampling offers a sense of empowerment and participation for one's health that is hard to achieve in clinical settings. It can be a socially and economically viable option to increase access to cervical screening for women not participating in routine screening. Unlike the findings in Brewer’s study among others, most Indigenous women in Moxham’s study were unwilling to use self-sampling for fear of administering it incorrectly, hurting oneself, or the need for re-screening if HPV is detected. Further research is needed to address this discrepancy.

In November, 2020, WHO launched the ”global strategy to accelerate the elimination of cervical cancer as a public health problem” programme, with the aim of eliminating cervical cancer by 2030. To achieve this goal in the Western Pacific region, it is important to ensure that resource-dependent and tailored programmes of screening and vaccination are planned and executed. We encourage more studies on cervical cancer prevention and treatment from the Western Pacific region, especially from underserved women, with the long-term goal of eradicating cervical cancer and reducing the social inequities across the region.

